# Supplementary material for: P-TRAP: a Panicle Trait Phenotyping tool
Source: BMC Plant Biol. 2013 Aug 29;13:122. doi: 10.1186/1471-2229-13-122 (PMC3848748; doi:10.1186/1471-2229-13-122)
Supplement: Additional file 7 — P-TRAP source code. The Netbeans project that contains the source code of the application. [file 1471-2229-13-122-S7.zip › javadoc.zip!/javadoc/org-bioalg-graphprocessor/org/bioalg/graphprocessor/EdgeModel.html]

EdgeModel (GraphProcessor)


JavaScript is disabled on your browser.


- Package
- Class
- Tree
- Deprecated
- Index
- Help

*org.bioalg.graphprocessor 1.0*

- Prev Class
- Next Class

- Frames
- No Frames

- All Classes

- Summary:
- Nested |
- Field |
- Constr |
- Method

- Detail:
- Field |
- Constr |
- Method


org.bioalg.graphprocessor

## Class EdgeModel

- java.lang.Object
- - org.bioalg.graphprocessor.EdgeModel

- ---

    

  ```
  public class EdgeModel
  extends java.lang.Object
  ```

  Similar to the `Edge` class but is using vertices instead of points.

  Author:
  :   faroq

- - ### Constructor Summary

    Constructors

    Constructor and Description

    `EdgeModel(Vertex vertex1,
    Vertex vertex2)`
  - ### Method Summary

    Methods

    | Modifier and Type | Method and Description |
    | --- | --- |
    | `Vertex` | `getVertex1()` |
    | `Vertex` | `getVertex2()` |
    | `void` | `setVertex1(Vertex vertex1)` |
    | `void` | `setVertex2(Vertex vertex2)` |
    | `java.lang.String` | `toString()` |

    - ### Methods inherited from class java.lang.Object

      `clone, equals, finalize, getClass, hashCode, notify, notifyAll, wait, wait, wait`

- - ### Constructor Detail


    - #### EdgeModel

      ```
      public EdgeModel(Vertex vertex1,
               Vertex vertex2)
      ```
  - ### Method Detail


    - #### getVertex1

      ```
      public Vertex getVertex1()
      ```


    - #### setVertex1

      ```
      public void setVertex1(Vertex vertex1)
      ```


    - #### getVertex2

      ```
      public Vertex getVertex2()
      ```


    - #### setVertex2

      ```
      public void setVertex2(Vertex vertex2)
      ```


    - #### toString

      ```
      public java.lang.String toString()
      ```

      **Overrides:**
      :   `toString` in class `java.lang.Object`


- Package
- Class
- Tree
- Deprecated
- Index
- Help

*org.bioalg.graphprocessor 1.0*

- Prev Class
- Next Class

- Frames
- No Frames

- All Classes

- Summary:
- Nested |
- Field |
- Constr |
- Method

- Detail:
- Field |
- Constr |
- Method
